# Understanding dentists’ management of deep carious lesions in permanent teeth: a systematic review and meta-analysis

**DOI:** 10.1186/s13012-016-0505-4

**Published:** 2016-10-19

**Authors:** Falk Schwendicke, Gerd Göstemeyer

**Affiliations:** Department for Operative and Preventive Dentistry, Charité Centre for Dental Medicine, Aßmannshauser Str 4-6, 14197 Berlin, Germany

**Keywords:** Attitudes, Dental, Decision-making, Evidence-based practice, Qualitative studies, Surveys

## Abstract

**Background:**

Increasing evidence supports selective/incomplete (SE) or stepwise (SW) instead of non-selective/complete tissue removal for deep carious lesions in vital teeth, mainly as pulpal risks are significantly reduced. Our aims were to analyze the proportion of dentists who utilize SE/SW for deep lesions in permanent teeth and to identify barriers and facilitators of utilizing SE/SW.

**Methods:**

We included studies that were original, and reported on the proportion of dentists utilizing SE/SW (quantitative studies), or reported on barriers or facilitators of such utilization (qualitative studies). Electronic databases (PubMed, CENTRAL, Embase, PsycINFO) were searched and screening and data extraction performed by two reviewers. Random-effects meta-analysis and meta-regression were used for quantitative synthesis of the proportion of dentists utilizing SE/SW. Thematic analysis was performed to assess barriers and facilitators on SE/SW utilization. Identified themes were translated into the constructs of the theoretical domains framework.

**Results:**

From 1728 articles, nine studies were included, all using quantitative methods. Four thousand one hundred ninety-nine dentists had been surveyed. The mean (95% CI) proportion of dentists using SE/SW for deep lesions was 53 % (44/62 %). More recent studies reported significantly higher proportions (*p* < 0.05). Reported estimates and thematic analysis found dentists’ age and an understanding of the disease caries and the scientific rationale behind different removal strategies to affect dentists’ behavior. Guidelines, peers, and the social and professional identity were further associated with the motivation of utilizing SE/SW. Environmental incentives, sanctions, or restrictions, mainly of financial but also regulatory character, impacted on decision-making, as did the specific indication (the patient, the tooth) and the beliefs on how well different treatments perform.

**Conclusions:**

Around half of all dentists rejected evidence-based carious tissue removal strategies. A range of factors can be addressed for improving implementation. Future studies should use mixed qualitative-quantitative methods to yield a deeper understanding of dentists’ decision-making.

**Trial registration:**

PROSPERO CRD42016038047

**Electronic supplementary material:**

The online version of this article (doi:10.1186/s13012-016-0505-4) contains supplementary material, which is available to authorized users.

## Introduction

Dental caries is a widely prevalent disease, burdening billions of individuals and causing significant healthcare costs [1, 2]. The treatment of deep carious lesions is especially challenging for dentists, as such lesions are cavitated, with some dentin removal being required prior to placing a restoration to ensure the longevity of the restoration [3], while such removal of dentin in proximity to the pulp is risky [4]. Increasing evidence supports selective (“incomplete”) or stepwise instead of non-selective (“complete”) dentin removal in deep lesions of permanent teeth, as both avoid pulp exposure and postoperative complications [3–5]. While non-selective removal uses the same assessment criterion everywhere in the cavity (thus leading to possibly harmful removal of dentin in proximity to the pulp), selective removal aims to leave hard dentin in the periphery and soft or leathery dentin in the pulpo-proximal areas of the cavity [1, 2]. Stepwise removal stands in-between, with the first step equaling selective removal, followed by a temporary sealing of the cavity, followed by re-opening some months later and non-selective removal [1]. While both stepwise and selective removal are recommended for treating deep lesions in permanent teeth, selective removal has some advantages over stepwise removal (i.e., a reduced risk of pulp exposure, fewer number of visits needed, and lower costs) [1, 2].

A number of survey studies, however, indicate that dentists have not widely adopted these less invasive, evidence-based strategies for managing deep lesions [6–9]. So far, it is unknown what the overall proportion of dentists who adopted less invasive carious tissue removal in different countries is and if this proportion has changed with time. Furthermore, it is unknown why dentists adopt or reject selective or stepwise carious tissue and what factors generally drive their decision-making towards deep lesions.

The objective of this systematic review was to analyze the proportion of dentists surveyed in different countries who employ selective (SE) or stepwise (SW) excavation of deep lesions in permanent teeth and to assess a possible change of this proportion with time. A further objective was to identify barriers and facilitators to adoption of SE/SW instead of non-selective excavation, which could assist tailoring implementation interventions. To reduce the risk of bias during the review process and yield more comprehensive, valid, and reliable results, a theory-based systematic assessment of barriers and facilitators was to be used [10].

## Methods

This review was registered a priori at PROSPERO (CRD42016038047) and was planned to utilize qualitative and quantitative data. However, the included studies were all quantitative (survey or poll design) and did not employ qualitative research. Consequently, and in deviation of the protocol, we used a different scale for risk of bias assessment. The reporting of this study is in accordance with Additional file 1, the PRISMA, and the ENTREQ statement guidelines [11, 12].

### Eligibility criteria

This systematic review included studies that were original and reported on the proportion of dentists utilizing one or the other carious tissue removal strategy and/or reported on barriers or facilitators of such utilization in adults or children with permanent, vital (sensible) teeth with deep carious lesions. No restriction as to the pulp symptomatology was defined a priori, but given the focus of our review only studies investigating the management of teeth where maintaining pulp vitality was an option were included. Studies investigating only the management of the exposed pulp were excluded, as were those investigating management of primary teeth only.

Included studies could be interviews, focus groups, surveys or studies using other observational designs, also if nested in a larger trial with different purpose. Only peer-reviewed publications were considered. No language, time, or quality restrictions were applied.

#### Outcomes

The outcome of this review was the utilization SE/SW instead of non-selective carious tissue removal. We assessed the proportion of dentists choosing one or both of these removal strategies for deep lesions as well as barriers or facilitators for such utilization.

### Information sources

#### Electronic searches

We searched Embase, Medline via PubMed, Cochrane CENTRAL, PsycINFO, and Google Scholar. We have not searched conference abstracts, as we assumed the information presented there to be too limited to allow synthesis. Dissertation and theses were searched via ProQuest and Dissertations and Theses. We additionally searched for reviews using the Database of Abstracts of Reviews of Effects (DARE), the Health Technology Assessment database (HTA), and NHS EED, as such reviews could have yielder further, so far not identified original studies. In addition, reference lists of identified full texts were screened and cross-referenced. We contacted study authors if required to obtain full texts or for clarification.

### Search strategy

The developed search strategy was as sensitive as possible given the expected limited indexing. The following strategy was used and individualized for each database: Search ((((((dentists) OR dentist) OR practitioner) OR practitioners)) AND (((deep) OR pulp) OR vital)) AND (((((caries) OR carious) OR cavity) OR lesion) OR decay).

### Study records

#### Selection process

A spreadsheet was used for data extraction and management. Both reviewers independently screened titles and compared findings. There was no disagreement. Full texts were assessed independently. No duplicative studies were assessed. Studies were included in agreement.

#### Data collection process

Data extraction was performed independently by both reviewers. There were no disagreements during extraction.

### Data items

The following items were collected: authors; year; study type and sampling and survey/interview method; characteristics of dentists being investigated (country and demographics); lesion scenarios; proportion of dentists using different removal strategies; knowledge, attitudes, and believes underlying dentists’ decision, as identified by the study itself, or as discussed by the study authors (see below).

### Data synthesis

#### Data synthesis

The meta-analysis of the proportion of dentists performing SE/SW per all surveyed dentists was performed using Comprehensive Meta-Analysis 2.2.064 (Biostat, NJ, USA). Heterogeneity was assessed using Cochrane’s *Q* and *I* ^2^-statistics [13]. Since heterogeneity was found high, a random-effect model was used. To explore temporal changes of the proportion, we performed meta-regression using the unrestricted maximum-likelihood method [14, 15]. Publication bias was assessed using funnel plots as well as Egger’s regression intercept test [16].

To synthesize qualitative data, we had planned thematic analysis of reported original qualitative data. Given that no qualitative study was included, we relied on reports as to how patient- or dentist-center factors (age, gender, caries risk, practice setting) impacted on decision-making towards deep lesions. We additionally screened the discussion of each manuscript for potentially relevant aspects; findings based on the study authors’ judgement were extracted and highlighted as such during the thematic analysis.

Factors possibly associated with dentists’ excavation behavior were independently abstracted by both authors. In an iterative approach of theme and category development, relationships between themes and concepts were independently identified by both reviewers [17]. Afterwards, reviewers compared their findings and jointly grouped and translated them into the domains and constructs of the TDF [18, 19]. The rationale for assignment of the barriers and facilitators to the TDF domains can be found in the Appendix (Additional file 2: Table S1). To improve the applicability of our findings for implementation, TDF domains were aligned with domains of the Behavior Change Wheel to facilitate the deduction of interventions [20]. The developed framework was double-checked by repeated coding and referencing from and to all included studies. Themes and concepts were further classified as barriers (−) or facilitators (+) of selective or stepwise excavation, or as conflicting, i.e., uncertain as to their effect on the target behavior (?) [18, 21, 22].

### Quality assessment and confidence in data

Quality assessment of included studies was based on the Newcastle-Ottawa Scale (NOS) for cross-sectional studies [23]. Note that we adapted the scale, using an item on questionnaire design, validity and reporting instead of the item on ascertainment of exposure, as the latter was not of relevance in the included studies. We judged studies with 0–3 of the maximum 8 NOS points (“stars”) to have high risk of bias, those with NOS 4-6 moderate and those with NOS 7 or 8 to have low risk of bias. Both reviewers independently assessed the quality of each study. There were no discrepancies during rating.

## Results

### Search and included studies

**Fig. 1:**
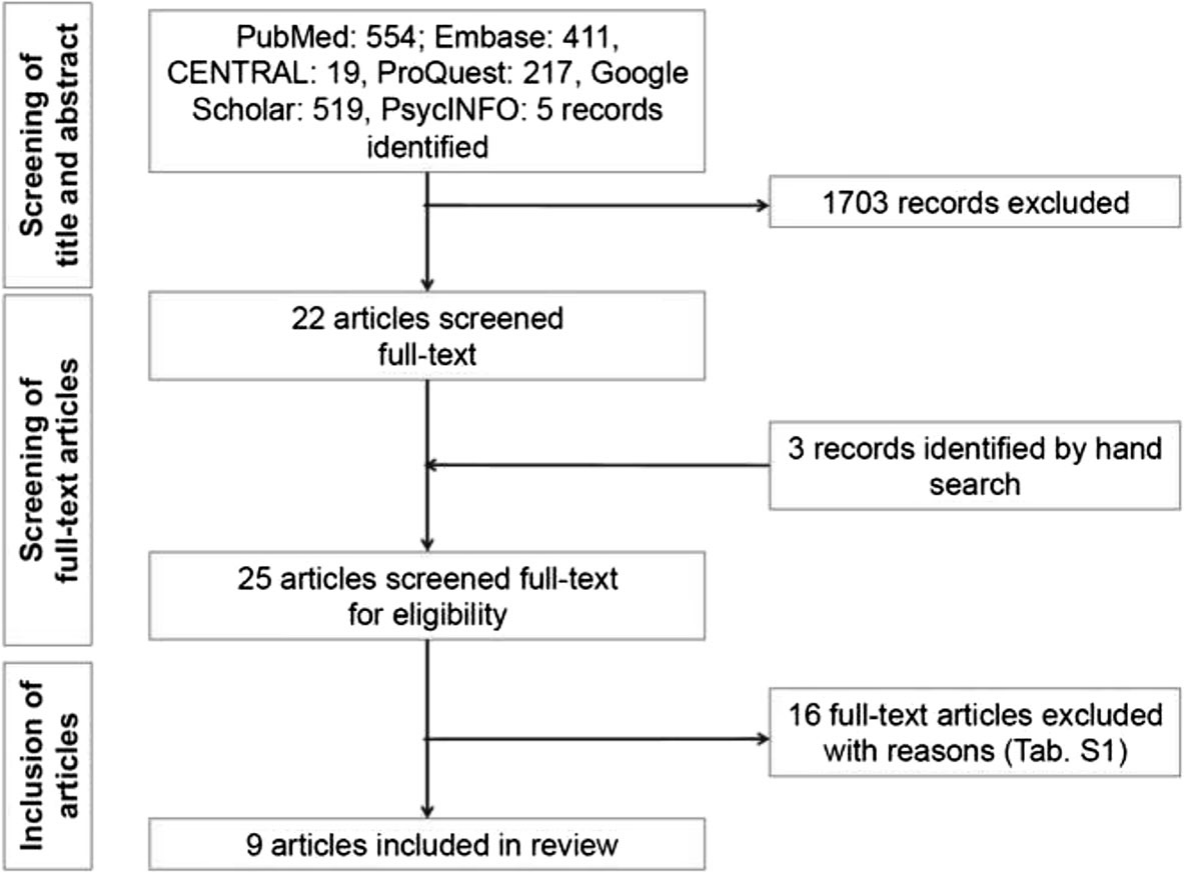
Flow of the search

We identified 554 articles via PubMed, 411 via Embase, 19 via Cochrane CENTRAL, 217 via ProQuest, 519 via Google Scholar, 5 via PsycInfo, and none via DARE or other screened databases. Three more articles were identified via cross-referencing. From these, 25 were screened in full-text and 9 included (Fig. 1). Studies were mainly excluded as they did not investigate the management of deep carious lesions in permanent teeth; one study was excluded as insufficient data were reported and authors could not be contacted. Details on excluded studies can be found in the appendix (Additional file 3: Table S2).

The nine included studies (Table 1) were published between 2007 and 2016. Eight studies used questionnaires and one study real-time polling for surveying. Sample sizes ranged between 54 and 1481 responders, with a response rate between 28 and 92 %. Overall, 4199 (3845 general, 354 specialized) dentists were involved. Six studies used a scenario comprising a deep carious lesion in a permanent tooth with either asymptomatic (four studies) or unknown (two studies) pulp status. The other three studies did not use a scenario (two studies) or did not clearly describe the scenario (one study). Participants could usually choose between non-selective (complete) and SE (partial, incomplete) caries removal. Six studies additionally allowed to choose between SW or endodontic treatment (direct capping, pulpotomy, root-canal therapy).

**Table 1:** Included studies

Study	Method	Country, year	Sample	Scenario	Treatment decisions	Reasons, barriers, facilitators
Oen [9]	Quest	USA, 2006	PEARL research network, response 92 %, final 85	Deep dentin lesion in permanent molar with vital (sensible) pulp (pain lasts <3 s) and different risk of exposure	No risk of exposure: 58/85 CR, 19/85 SE, 8/85 endodontic treatment Risk of exposure: 51/85 CR, 15/85 SE, 17/85 endodontic treatment	Age and general caries risk/experience of patient influenced decisions (more invasive in older and high-risk patients); dentists knew risk of failure of direct capping. Authors discuss peer pressure and educational background.
Seale and Glickman [29]	Real-time poll	USA, 2007	Conference, 376 dentists (102 endodontists, 252 pedodontists, 22 others), unclear response	Young permanent teeth with open apex	Pedodontists: 179/252 SW, endodontists: 222/376 SW	Reasons against SW: second visit needed—compliance problems, MTA pulpotomy better evidence, SE better evidence, reimbursement. Reasons for SW: option to recall symptoms/vitality, root maturation, low costs, re-assess dentin, payment for second appointment.
Weber [8]	Quest	Brazil, 2009	Dentists from one southern city 44 % response, final 54	Deep carious lesion in permanent molar with vital pulp and no spontaneous pain, but pain when chewing or cold	42/53 CR, 7/53 SW, 4/53 SE. We excluded the third case as the pulp and peri-apical status were unclear.	SE/SW: female OR 0.6 (0.2–1.2), younger (graduation >2000): OR 5.5 (1.5–19.7), possible reasons: SW requires second appointment, patients, do not return, younger dentists use evidence-base better.
Chisini [34]	Quest	Brazil, 2009	Single city, all dentists, 68 % response, final 187	Deep lesion in proximity of pulp, unclear pulp status and dentition (assumed permanent)	65/171 CR, 106/171 SE	Dentists with more recent graduation or postgraduate training chose SE more often. Authors evaluated experience and setting (public versus private practice versus university).
McBride [35]	Quest	USA, 2009/2010	National, practice based research networks, 66–82 % response, final 950	Lower molar with visible cavitated lesion, deeper than anticipated, may involve pulp (pulpal status not stated)	372/812 CR 285/812 SE 155/812 ET	Age was found a factor, with dentists practicing 5–15 years performing ET more often, while those <5 years performed SE more often; full network participant also more likely to perform SE.
Stangvaltaite [7]	Quest	Norway, 2011	Northern Norway, all dentists, 56 % response, final 222	Deep carious lesion in permanent mature teeth without symptoms and exposure (further scenarios: with symptoms and exposure)	Without symptoms and exposure: 104/212 CR, 95/212 SW, 13/212 SE	CR versus SE: male OR 1.5 (0.8–2.8), from Norway: 0.5 (0.2–0.9), public practice: 0.6 (0.3–1.3), experienced (5+ years): 1.3 (0.7–2.6), urban: 2.2 (1.2–4.1), main reasons for choosing a strategy were good results, easy, restoration longevity, patients’ health; SW recommended in guidelines.
Katz [27]	Quest	Brazil, 2012	Northeastern Brazil, participants of a regional dentistry congress, final 123	Unclear scenario	59/108 CR, 49/108 SE	Majority of dentists considers caries to be treated only restoratively. Attitudes towards minimally invasive dentistry procedures significantly associated with SE (professionals considering minimal invasive as permanent recommended SE); Lack of belief in SE rather than knowledge or specialist status drove decision-making.
Schwendicke [28]	Quest	Germany, 2012	Northern Germany, all practitioners, 35 % response, final 821	Young female patient with deep lesion in vital asymptomatic tooth, risk of pulp exposure	400/799 CR, 160/799 SE, 239/799 both	Dentists aware of risks and success rates; dentists who accepted bacteria to remain and possible restorative risks were more likely to SE, those who strived for restorative longevity and feared bacteria to remain performed CR and accepted ET. Demographics not a factor; generally more or less invasive dentist types.
Schwendicke [6]	Quest	Germany, France, Norway 2015	National, all practitioners, 28–50 % response, final 1481	Deep lesion in permanent tooth with a vital painless pulp with risk of exposure in young patient	France: 340/661 CR, 62/661 SE, 259/661 SW, Germany: 201/622 CR, 122/622 SE, 299/622 SW, Norway: 3/199 CR, 29/199 SE, 167/199 SW	Male dentists chose SE more often (OR: 1.73 [1.26/2.45]), dentists in private setting performed fewer SW (0.60 [0.39/0.93]), those who believed bacteria needed removal to avoid progression chose SE less often (0.48 [0.33/0.71]), as did those who feared bacteria to harm the pulp (0.42 [0.28/0.62]) and vice versa for those who thought sealed lesions to arrest (2.84 [1.86/4.36]) or who strived to avoid exposure (2.18 [1.40/3.29]). Satisfaction with a treatment, familiarity and its evidence-base were main reasons, only few stated financial issues or peers as problems, knowledge also minor factor. Authors discuss education, caries philosophy as further reasons.

The proportion of dentists performing selective (SE), stepwise (SW), “complete” removal (CR), or immediate endodontic treatment (ET) for different scenarios of deep lesions were assessed. In addition, reasons (barriers, facilitators) for the decisions were recorded

### Study quality assessment

Risk of bias of included studies is shown in Table 2. Most of the included studies were found to have yielded representative samples of dentists. This sample, however, had not always been drawn nationally. Not one study reported on a sample size calculation, and most had significant non-response rates, which were not accounted for sufficiently by all but two studies. The validity of the survey was not described or demonstrated by most studies, and in five studies, the survey instrument was not published. Given the design of all studies, outcomes were only self-reported, which impacts on credibility of the findings. Last, statistical evaluation was not accounting for confounders appropriately. Overall risk of bias was high in four studies [8, 9, 27, 29], moderate in four studies [6, 7, 34, 35], and low in one study [28].

**Table 2:** Risk of bias according to the modified Newcastle-Ottawa Scale for cross-sectional studies [23]

Item	Oen [9]	Seale and Glickman 2007 [29]	Weber [8]	McBride [35]	Stangvaltaite [7]	Schwendicke [28]	Chisini [34]	Schwendicke [6]	Katz [27]
Selection									
Representativeness of the sample	*		*	*	*	*	*	*	*
Sample size determination									
Non-responders				*		*			
Validity of survey									
Checked reliability and internal consistency Survey available				**		**	*	**	
Comparability	*	–	–	–	*	*	*	*	–
Outcome									
Assessment of the outcome	*	*	*	*	*	*	*	*	*
Statistical test			*		*	*		*	
Overall	***	*	***	*****	****	*******	****	******	**

For each risk of bias domain, one to two stars could be collected, with a total number of eight stars being possible. We classified studies as high risk (1–3 stars), moderate risk (4–6 stars), or low risk (7–8 stars)

### Meta-analysis

**Fig. 2:**
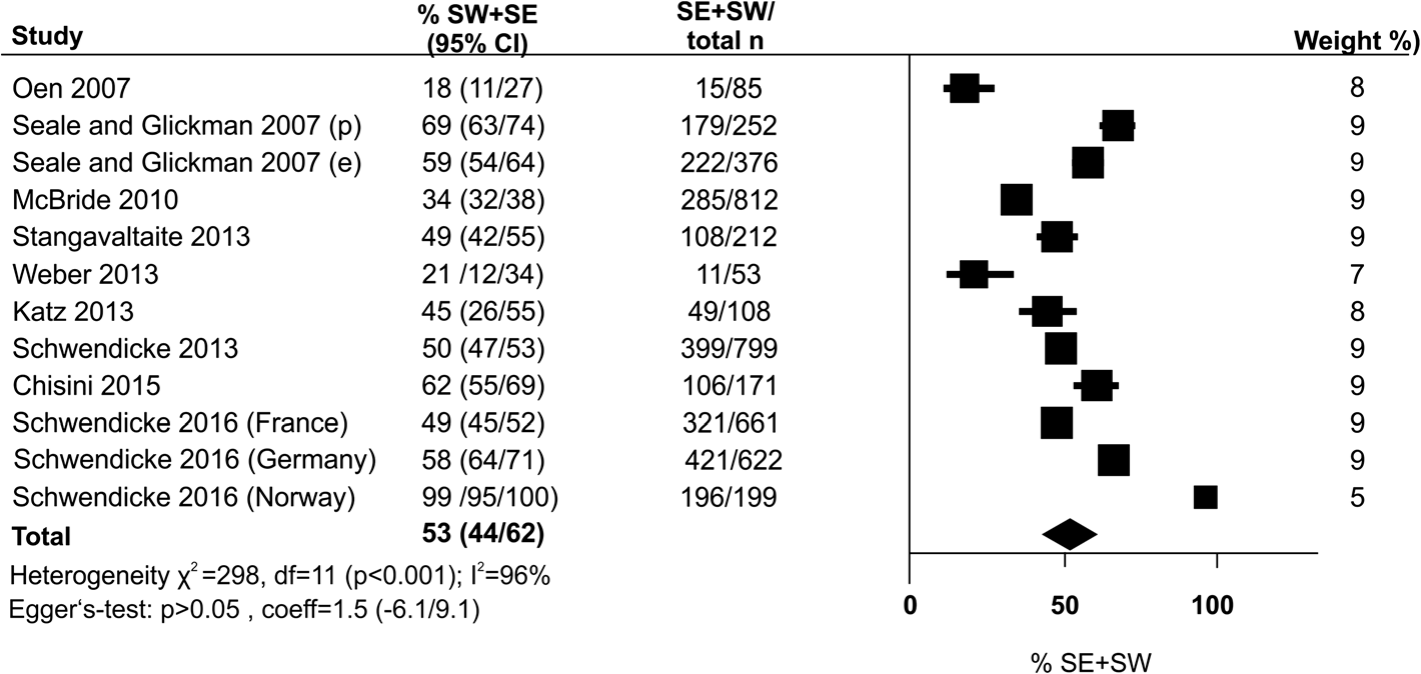
The proportion of dentists who performed selective (SE) or stepwise (SW) carious tissue removal (% SW + SW). Wherever possible, subgroups of dentists (according to specialization like pedodontics [p] or endodontics [e], or in different countries) were separately entered into meta-analysis. The pooled proportion and 95 % confidence intervals (*bold*) from random-effects meta-analysis is shown as *diamond*. Heterogeneity was assessed using *χ* ^2^-test and *I* ^2^-statistics. Publication bias or small-study effects were evaluated using Egger’s regression intercept test as well as funnel plot analysis. n total sample size

**Fig. 3:**
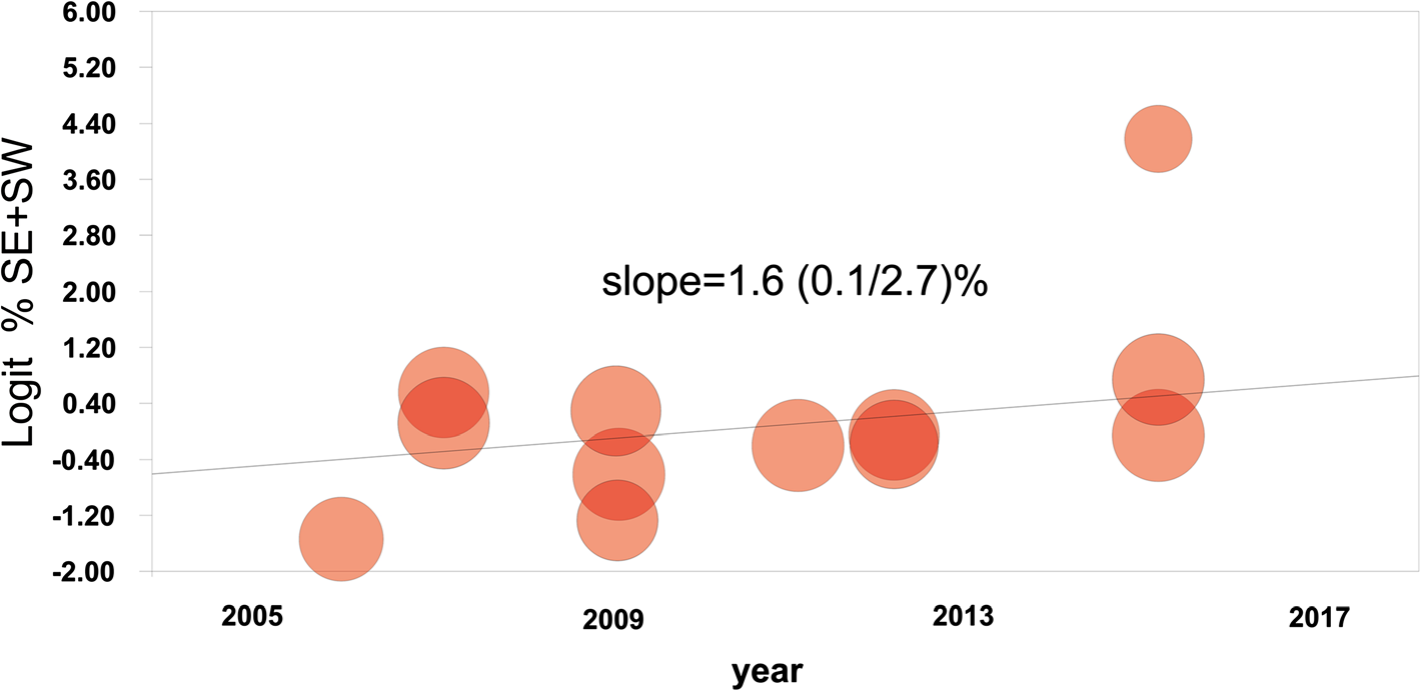
Association between the year of study publication and the share of dentists performing selective (SE) or stepwise (SW) carious tissue removal. Every circle is the weighted estimate of each study. The regression line indicates a significantly increased share in recent years (*p* = 0.048), with a mean (95% CI) slope of 1.6 (0.1/2.7 %), i.e., the share increased with 1.6 % per year in mean

**Fig. 4:**
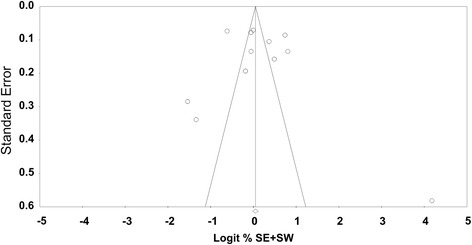
Funnel plot. Standard errors are plotted against the logarithm of the share of dentists performing selective (SE) or stepwise (SW) carious tissue removal. No significant asymmetry was identified

All nine studies contributed to meta-analysis (Fig. 2). In five studies, the majority of participants chose SE/SW for deep lesions. Overall, the mean (95% CI) proportion of dentists using SE/SW was 53 % (44/62 %). This was significantly associated with the year of study conduct (Fig. 3), with an increasing proportion in later studies (+1.6 % per year). There was no indication for publication bias via statistical or graphical analysis (Fig. 4).

### Factors associated with carious tissue removal behavior

**Fig. 5:**
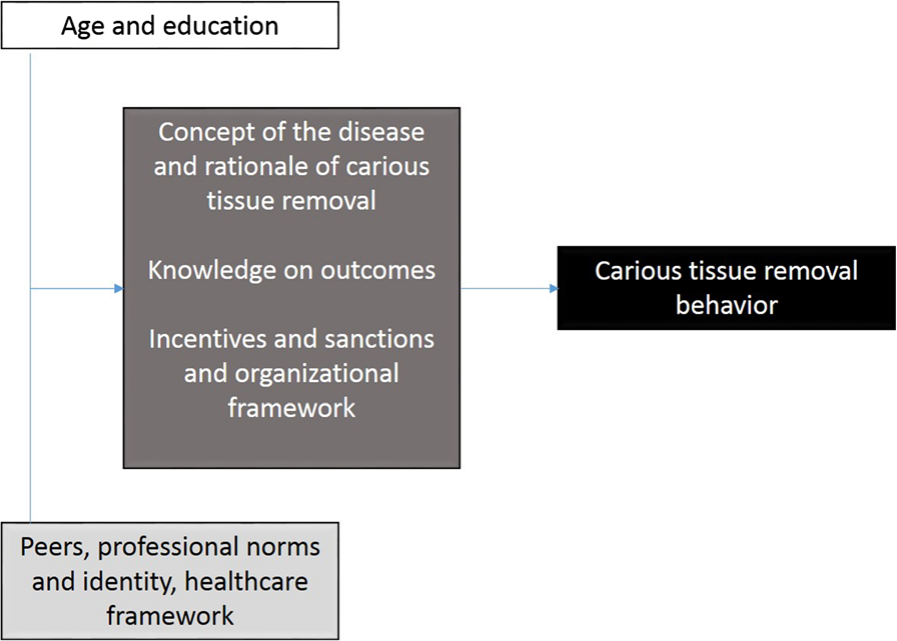
Identified factors shaping dentists’ carious tissue removal behavior according to the domains of the Behavior Change Wheel [20]. Capability (*white box*) is shaped by dentists’ education, which in turn differs between younger and older dentists. Opportunity (*light grey box*) is influenced by peers and associated professional norms and identities as well as healthcare organization. Both capability and opportunity shape dentists’ motivation (*dark grey box*), which is affected by the understanding of the disease caries and the rationale of carious tissue removal, knowledge on the outcomes (of different removal strategies, but also endodontic therapies), and healthcare incentives or sanctions. All factors eventually guide the carious tissue removal behavior (*black box*)

Identified barriers or facilitators or conflicting themes were mapped to a number of TDF domains and constructs, mainly on knowledge, social influence, environmental context, beliefs, and reinforcement (Table 3). These covered all aspects of the Behavioral Change Wheel domains. We found dentists’ age to affect knowledge, with an understanding of the disease caries and the scientific rationale behind different excavation strategies eventually affecting behavior. Guidelines, peers, and the social and professional identity of dentists further were associated with the motivation of utilizing behaviors SE/SW. Environmental incentives, sanctions, or restrictions, mainly of financial but also regulatory character, impacted on decision-making, as did the specific indication (the patient, the tooth) and the beliefs on how well different treatments perform. It should be noted that this outcome expectancy was not always related with the chosen excavation strategy. The possible interactions between different domains of the Behavioral Change Wheel (namely, capability, opportunity, and motivation) are summarized in Fig. 5.

**Table 3:** Mapping of identified themes to COM-B (capability, opportunity, motivation) of the Wheel of Change [36] and TDF domains and constructs [19]

COM-B domain	TDF domain	TDF construct	Identified enabler (+) or barrier (−) or conflicting theme (?)	Explanation	Reference
Capability	Knowledge	Knowledge of condition, scientific rationale	(−) age (younger dentists more likely to perform SE or SW) (+) understanding of caries (acceptance of remaining bacteria being sealed)	Younger dentists have different knowledge on caries and the rationale of carious tissue removal.	[6–9, 27–29, 34, 35]
	Skills	Procedural knowledge, skills, competence, ability	(+) dentists oftentimes adopt to new techniques (liners, burs etc.)	Many dentists are adopters of technical change; skills are not a barrier for different carious tissue removal.	[6–9, 27, 29, 34]
Opportunity	Social influence	Social pressure, norms, support, modelling	(−) peers (+) being part of a practice network	Fears of peers not accepting SE or SW are barriers. A practice network drives a different group dynamics and facilitates change.	[6, 9, 35]
	Social role	Professional identity, confidence	(?) gender (most studies found female dentists choosing SE or SW more often)	Male dentists might have different professional identity which could act as barrier.	[6, 7, 29]
	Environmental context and resources	Stressors, resources, organizational culture	(−) financial aspects, private practice model associated with more invasive treatments (+) presence of guidelines (e.g. for stepwise in Norway) (?) healthcare organization (significant between-country differences)	Being paid for quantities of treatment sets the incentive to treat, not to maintain pulp vitality. Such incentive was especially found in private practices (fee for item reimbursement). Reimbursement and regulation in different countries could lead to observed between-country differences. Having guidelines towards less invasive excavation facilitates change.	[6, 7, 28, 29]
Motivation	Beliefs about capabilities	Self-confidence, competence, control	(−) education, role of the dentists as perceived as expert	Dentists see themselves as experts. The acquired education is a firm foundation for their beliefs, which could act as barrier.	[7, 29, 34]
	Beliefs about consequences	Outcome expectancies	(?) knowledge on expected outcomes (?) patient or tooth specific expectations (−) compliance needed in SW	The expected outcome might drive some decisions (decisions are tailored to teeth or patients based on different expectations). However, expectations are not always predicting decisions.	[8, 9, 27–29, 34]
	Reinforcement	Rewards, incentives	(−) financial aspects, practice settings	See above.	[7, 28, 29, 34]
		Sanctions, punishment	(−) healthcare organization (country-specific, guarantee times for restorative)	See above.	[6, 28, 29]
	Memory attention and decision process, optimism	Decision process, pessimism	(−) compliance needed in SW	See above.	[7, 8, 29]

## Discussion

This review did not identify qualitative studies on dentists’ decision-making with regards to deep carious lesions; we only included quantitative observational studies. The reported barriers and facilitators for utilizing SE/SW have been extracted from these quantitative studies. It is clear that this was unlikely to yield a saturation of themes and concepts, which calls for the conduct of an in-depth study using for example interviews or focus group discussions to yield a deeper understanding of barriers and facilitators on this topic.

We found dentists to rely heavily on invasive strategies for treating deep lesions in permanent teeth: around half of all surveyed dentists preferred non-selective removal or even immediate endodontic treatment for vital teeth, i.e. those where maintaining pulp vitality is—at least in theory—still possible. Such high proportion of unnecessarily invasive interventions is worrisome, as growing evidence indicates that non-selective excavation or the immediate sacrifice of pulp vitality is likely to reduce the retention time of the tooth, burdening the patient and generating significant treatment costs [24–26]. It was noteworthy that more recent surveys yielded a higher proportion of dentists utilizing SE or SW; however, this trend was only borderline significant (which might have to do more with statistical power than a lacking association) and could be an artifact given that dentists in different countries were surveyed. However, data from Germany confirmed this trend, where a representative sample of dentists from Northern Germany had been surveyed in 2013, with 49 % considering SE/SW [28], while this number increased to 68 % in a national sample in 2016 [6].

Using thematic analysis and mapping the identified themes to constructs of the TDF allowed us to understand a number of aspects which were associated with dentists’ decision-making for deep lesions. One relevant domain was knowledge; however, several studies found that knowledge on expected outcomes (success rates of different strategies) did not necessarily predict decision-making [27–29]. Instead, an understanding of the disease caries and the associated scientific rationale behind different excavation strategies seemed more important: dentists who understood caries as infectious disease and who feared remaining bacteria to harm the pulp preferred non-selective excavation more frequently. It was further assuring to find younger dentists to be less invasive than older dentists in most studies; something which we ascribe to a change in education concepts. Future studies should engage into understanding the impact of gender, as most but not all studies found male dentists to be more invasive than female dentists.

A second factor was the context of the decision: Specific patient or tooth level aspects (patient’s age, tooth maturity, type of exposure) seemed to guide decision-making, again something which is likely to relate to knowledge, and could be tackled by future implementation interventions. The presence (or absence) of professional guidelines was stated as one facilitator (or barrier) for performing SE/SW. The opinion of peers towards different removal strategies seems to impact (with presumably different peer pressure in practice research networks, for example). Healthcare regulations and incentives or sanctions (financial gain from different treatments, sanctions on restorative complications) were further aspects.

This study has a number of strengths and limitations. First and as discussed, our qualitative findings are relatively limited and unlikely to represent all relevant themes. The use of a theoretical base for understanding these aspects, however, strengthens the credibility of our findings, as the TDF is a comprehensive and validated instrument. The constructed link to the Behavior Change Wheel further supports the use of our data for studies on implementation interventions. Second, we needed to make some assumptions for assigning barriers or facilitators to the TDF domains. For example, we assumed that a specific “education of the dentist” might act as a barrier when considering it from the TDF domain “self-confidence, competence, and control.” That was done, as we assumed that dentists, confidently perceiving themselves as the main experts on caries treatment, might be reluctant to adopt changes to their daily practice. This assumption (and others) might be disputable; this uncertainty when assigning themes to TDF domains should be born in mind when interpreting our findings. Third, the quantitative aspects of our study are built on a limited number of studies; this limited number might be the result of the applied inclusion criteria. In this sense, we could have considered conference abstracts for inclusion, and could have contacted abstract authors for more detailed information. The included studies were additionally very heterogeneous. For example, sample sizes, sampling methods, survey instruments, and constructed scenarios differed widely, which probably contributed to the observed statistical heterogeneity. That was also the reason why we did not attempt to pool data on barriers and facilitators, which were additionally scarce. Moreover, only six countries were assessed in total (some several times), which prohibits generalization of our findings. The included studies were all surveys or polls by design, i.e., yielded self-reported data, and had further qualitative weaknesses (mainly related to sampling, non-response, and the used instrument). The associated risk of bias is likely to lead to some distortions (via the Hawthorne effect or selection bias, for example). Last, we only assessed decision-making in permanent teeth, while a number of studies investigated dentists’ behavior in primary teeth [30, 31]. Given the clinical decisions to be made and their consequences being very different in primary versus permanent teeth, we had decided to not include studies on primary teeth.

A number of recommendations can be deduced from this study. Future research in this direction should involve qualitative elements to yield a deeper understanding of barriers and facilitators of utilizing SE/SW. When using a less rigid format than a predefined survey, new themes and concepts are likely to emerge. Possible interventions on increasing the proportion of dentists performing SE/SW instead of non-selective excavation should focus on improving the understanding of the disease caries and the rationale behind different excavation strategies [32, 33] and should aim to eliminate barriers for evidence-based management of deep lesions on both practice and healthcare level.

## Conclusions

Nearly half of all dentists sampled by the included studies preferred invasive instead of evidence-based management strategies for deep carious lesions in permanent teeth. In recent years, this proportion seems to decrease. Dentists’ behavior was affected by a range of factors, from their understanding of what constitutes caries and how it should be managed in general, over contextual factors to systemic (oftentimes economic) reinforcement mechanisms. Given only quantitative studies being included in this review, future studies should involve some qualitative elements to yield a deeper understanding of barriers and facilitators towards less invasive carious tissue removal. Such understanding would also be needed for tailoring implementation of interventions.

## Additional files

Additional file 1: PRISMA 2009 checklist. (DOC 63 kb)
Additional file 2: Table S1. Rationale for assignment of different identified enabler (+), barrier (−), or conflicting (?) themes to the TDF domains. (DOC 34 kb)
Additional file 3: Table S2. Excluded studies. (DOC 76 kb)

## Abbreviations

COM-B: Capability, opportunity, motivation and behavior model
CR: Complete removal of caries
ET: Endodontic treatment
NOS: Newcastle-Ottawa Scale
SE: Selective excavation
SW: Stepwise excavation
TDF: Theoretical domains framework
