# Supplementary material for: Understanding dentists’ management of deep carious lesions in permanent teeth: a systematic review and meta-analysis
Source: Implement Sci. 2016 Oct 19;11:142. doi: 10.1186/s13012-016-0505-4 (PMC5069935; doi:10.1186/s13012-016-0505-4)
Supplement: Additional file 2: — Table S1. Rationale for assignment of different identified enabler (+), barrier (−), or conflicting (?) themes to the TDF domains. (DOC 34 kb) [file 13012_2016_505_MOESM2_ESM.doc]

Additional file 2: Table S1: Rationale for assignment of different identified enabler (+), barrier (-) or conflicting (?) themes to the TDF domains.

| **TDF construct** | **Identified enabler (+) or barrier (-) or conflicting theme (?)** | **Rationale** |
| --- | --- | --- |
| Knowledge of condition, scientific rationale | (-) age (younger dentists more likely to perform SE or SW)  (+) understanding of caries (acceptance of remaining bacteria being sealed) | Older dentists perform SE or SW less often. We ascribe this to their education in cariology, which was based on a different understanding of caries and the rationale behind SE/SW. It was shown that knowledge of the condition caries and the rationale of SE/SW acts as facilitator for adoption of SE/SW. |
| Procedural knowledge, skills, competence, ability | (+) dentists oftentimes adopt to new techniques (liners, burs etc.) | Dentists have been found to adopt new procedural techniques for treatment readily. We assume the required procedural knowledge and skills to act as facilitators for adoption of SE/SW. |
| Social pressure, norms, support, modelling | (-) peers  (+) being part of a practice network | The impact of peers and the associated social pressure was shown to act as barrier for adoption of SE/SW.  Moreover, dentists who were members in practice networks are submitted to a different time of norm setting/modelling as well as social support. That has been shown to act as facilitator for adoption of SE/SW. |
| Professional identity, confidence | (?) gender (most studies found female dentists choosing SE or SW more often) | Gender was – ambiguously – found to affect adoption of SE/SW. We ascribed this to a possibly different different professional identity in different genders. It should be highlighted that this assignment is highly subjective, as gender might also be associated with many other TDF domains (knowledge, skills). |
| Stressors, resources, organizational culture | (-) financial aspects, private practice model associated with more invasive treatments  (+) presence of guidelines (e.g. for stepwise in Norway)  (?) healthcare organization (significant between-country differences) | The financial setting and practice model has been shown to affect adoption of SE/SW, acting as a stressor due to resource guidance.  The presence of guidelines sets the organizational culture, the absence of supporting guidelines is acting as a stressor and barrier to adopting SE/SW. In general, the healthcare organizations and the associated organizational framework and culture affects adoption of SE/SW. |
| Self-confidence, competence, control | (-) education, role of the dentists as perceived as expert | The specific education of dentists was assumed to impact on the perceived role as an expert. The associated self-control, self-confidence and presumed competence was assumed to possibly act as barrier for adoption, as an established treatment concept, based on the perceived role as sole experts, decreases the readiness for change. |
| Outcome expectancies | (?) knowledge on expected outcomes  (?) patient or tooth specific expectations  (-) compliance needed in SW | The knowledge on the outcomes of SE/SW, i.e. the outcome expectancies, were assumed to affect adoption of SE/SW. One subcomponent are the expectancies of patients, or the specific expectancies for certain teeth (e.g. primary versus permanent teeth). Another component of outcome expectancies towards SW is the issue of patients’ compliance being needed. A presumed limited compliance will negatively affect outcome expectancies and act as barrier. |
| Rewards, incentives | (-) financial aspects, practice settings | Besides the above assignment of financial aspects, we also assigned it to the domain rewards/incentives. |
| Sanctions, punishment | (-) healthcare organization (country-specific, guarantee times for restorative) | Besides the above assignment of healthcare organization, we also assigned it to the domain sanctions/punishment. |
| Decision process, pessimism | (-) compliance needed in SW | Besides the above assignment of compliance, we also assigned it to the pessimism. |
