# Supplementary material for: Understanding dentists’ management of deep carious lesions in permanent teeth: a systematic review and meta-analysis
Source: Implement Sci. 2016 Oct 19;11:142. doi: 10.1186/s13012-016-0505-4 (PMC5069935; doi:10.1186/s13012-016-0505-4)
Supplement: Additional file 3: — Table S2. Excluded studies. (DOC 76 kb) [file 13012_2016_505_MOESM3_ESM.doc]

Additional file 3: Table S2: Excluded studies

| **Study** | **Reason for exclusion** |
| --- | --- |
|  | No treatment of deep lesions |
|  | No treatment of deep lesions |
|  | No treatment of deep lesions |
|  | No treatment of deep lesions |
|  | No treatment of deep lesions |
|  | No treatment of deep lesions |
|  | No treatment of deep lesions |
|  | No treatment of deep lesions |
|  | No treatment of deep lesions |
|  | Unable to extract required data* |
|  | No treatment of deep lesions |
|  | No treatment of deep lesions |
|  | No treatment of deep lesions |
|  | No treatment of deep lesions |
|  | No treatment of permanent teeth |
|  | No treatment of deep lesions |

* Could not contact study authors as no contact option available.

**References**

1. Frisk F, Kvist T, Axelsson S, Bergenholtz G, Davidson T, Mejare I, Norlund A, Petersson A, Sandberg H, Tranaeus S, Hakeberg M: **Pulp exposures in adults--choice of treatment among Swedish dentists.** *Swed Dent J* 2013, **37:**153-160.

2. Bjørndal L, Laustsen MH, Reit C: **Root canal treatment in Denmark is most often carried out in carious vital molar teeth and retreatments are rare.** *International Endodontic Journal* 2006, **39:**785-790.

3. Dobloug A, Grytten J, Holst D: **Dentist-specific variation in diagnosis of caries - a multilevel analysis.** *Community Dent Oral Epidemiol* 2014, **42:**185-191.

4. Elderton RJ: **Diagnosis and treatment of dental caries: the clinicians' dilemma. Scope for change in clinical practice.** *Journal of the Royal Society of Medicine* 1985, **78:**27-32.

5. Udoye CI, Sede MA, Jafarzadeh H, Abbott PV: **A survey of endodontic practices among dentists in Nigeria.** *J Contemp Dent Pract* 2013, **14:**293-298.

6. Palmer NO, Ahmed M, Grieveson B: **An investigation of current endodontic practice and training needs in primary care in the north west of England.** *Br Dent J* 2009, **206:**E22; discussion 584-585.

7. McCaul LK, McHugh S, Saunders WP: **The influence of specialty training and experience on decision making in endodontic diagnosis and treatment planning.** *Int Endod J* 2001, **34:**594-606.

8. Tan PL, Evans RW, Morgan MV: **Caries, bitewings, and treatment decisions.** *Aust Dent J* 2002, **47:**138-141; quiz 182.

9. Brennan DS, Spencer AJ: **Practice profiles of Australian private general dental practitioners.** *Aust Dent J* 2006, **51:**91-93.

10. Mayer R: **[Measures for the preservation of pulp vitality--relation to the age of the attending dentist].** *Zahnärzteblatt Baden-Württemberg* 1981, **9:**290-293.

11. Maidment Y, Durey K, Ibbetson R: **Decisions about restorative dental treatment among dentists attending a postgraduate continuing professional development course.** *Br Dent J* 2010, **209:**455-459.

12. Domejean-Orliaguet S, Leger S, Auclair C, Gerbaud L, Tubert-Jeannin S: **Caries management decision: influence of dentist and patient factors in the provision of dental services.** *J Dent* 2009, **37:**827-834.

13. Farag A, van der Sanden W, Mulder J, Creugers N, Frencken J: **Clinical Strategies for Managing Dental Caries in Egypt: Opinions of General Practitioner Dentists** *British Journal of Medicine and Medical Research* 2015, **5:**924-932.

14. Inglehart MR, Peters MC, Flamenbaum MH, Eboda NN, Feigal RJ: **Chemomechanical caries removal in children: an operator's and pediatric patients' responses.** *J Am Dent Assoc* 2007, **138:**47-55.

15. Fukai K, Ohno H, Blinkhorn A: **A cross-sectional survey investigating care of the primary dentition by paediatric dental specialists in Japan and the UK.** *Int Dent J* 2012, **62:**203-207.

16. Gordan VV, Bader JD, Garvan CW, Richman JS, Qvist V, Fellows JL, Rindal DB, Gilbert GH: **Restorative treatment thresholds for occlusal primary caries among dentists in the dental practice-based research network.** *J Am Dent Assoc* 2010, **141:**171-184.
